# Editorial: Adapted sports: Wheeled-mobility, exercise and health

**DOI:** 10.3389/fresc.2022.1015179

**Published:** 2022-09-23

**Authors:** Riemer J.K. Vegter, DirkJan H.E.J. Veeger, Vicky L. Goosey-Tolfrey, Christof A. Leicht

**Affiliations:** ^1^ Center for Human Movement Sciences, University of Groningen, University Medical Center Groningen, Groningen, Netherlands; ^2^ Peter Harrison Centre for Disability Sport, School of Sport, Exercise / Health Sciences, Loughborough University, Loughborough, United Kingdom; ^3^ Department of Biomechanical Engineering, Delft University of Technology, Delft, Netherlands

**Keywords:** wheeled mobility, paralympic sport, upper body, overuse injuries, wheelchair propulsion, kinetics, kinematics, classification

**Editorial on the Research Topic** Adapted sports: wheeled-mobility, exercise and health by Vegter RJK, Veeger DHEJ, Goosey-Tolfrey VL and Leicht CA. (2002) Front. Rehabilit. Sci. 3: 1015179. doi: 10.3389/fresc.2022.1015179

## Introduction

Persons that use a manual wheelchair depend on their upper body for daily mobility as well as for the sports they participate in. Numerous adapted sports exist. In some sports, modified wheelchairs are used for propulsion (e.g., wheelchair tennis, basketball, rugby and racing), others rely on other forms of cyclic upper-body exercise, like arm cranking movements (e.g., handcycling). Different adapted sports can also have an important impact on the upper body without a wheelchair involved, such as archery, paracanoe or swimming. What differentiates the abovementioned adapted sports from most able-bodied sports is the focus on the upper body for propulsion, which may result in different biomechanical and physiological responses when compared with the lower body.

The current Research Topic of Frontiers in Rehabilitation Sciences focuses on the performance and health aspects of participating in adapted sports and exercise for manual wheelchair users. 68 authors contributed to this special issue with 15 articles. They are spanning three broad topic areas:

(1) Shoulder-related responses and injuries resulting from upper-body exercise and wheelchair propulsion.
(2) Applied wheelchair sport research on wheelchair propulsion kinetics and kinematics.
(3) Elite sport and performance, considerations related to Paralympic sports specific classification.

## Shoulder-related responses and injuries resulting from upper-body exercise and wheelchair propulsion

The studies in this topic area put a spotlight on the shoulder as an anatomical structure that must cope with the very specific strains experienced during upper-body exercise, and more specifically, wheelchair propulsion. They provide new knowledge regarding activities that may be particularly stressful to the shoulder and surrounding structures and highlight factors that may help reduce strain, and as a result, minimise the risk for shoulder injury.

Bossuyt et al. investigated the acute shoulder tendon adaptations following maximal exercise in wheelchair rugby athletes. They provide evidence for exercise-mediated fluid inflow into the tendon, possibly because of the overload and acute inflammation. Arnet et al. demonstrated the benefits of fitness (assessed as anaerobic capacity) in the stabilisation of the shoulder during lifts, as it helped maintain the acromio-humeral distance following lifting. Aissaoui and Gagnon performed wheelchair propulsion training with haptic biofeedback with the aim to increase mechanical effectiveness and found the tangential push-rim force component to increase substantially, whilst also slightly increasing shoulder moments. Chénier et al. investigated sprinting with and without dribbling in wheelchair basketball, reporting higher speeds and shoulder loads when sprinting without dribbling. Finally, Mayrhuber et al. present a scoping review on shoulder injuries in wheelchair tennis. They identify possible risk factors as overhead movements, repetitive activation of the anterior muscle chain and internal rotators, as well as a higher spinal cord injury level.

## Applied wheelchair sports research on wheelchair propulsion kinetics and kinematics

Wheelchair sports and disability characteristics come in many shades, which results in a wide range of movement patterns. Kinetic and kinematic analysis allows to quantify impacts of equipment setup, practice and training interventions, with the aim to improve performance and avoid injury.

Three studies in this topic area investigate wheelchair-sport specific skills. Alberca et al. compared the impact of holding a badminton racket on wheelchair propulsion, reporting patterns associated with reduced propulsion effectiveness and higher injury risk. De Klerk et al. investigated wheelchair racing propulsion acquisition skills during three weeks of wheeling practice, reporting pronounced improvements in metabolic strain, push and cycle times. In a systematic review, Altman et al. identify tests for throwing maximal distance, throwing precision, and dribbling the ball to determine ball-handling proficiency in wheelchair sports.

A systematic review by Fritsch et al. outlines the methodologies used to study the impact of manual wheelchair configuration on biomechanical outcome measures. An applied example of a study using such methods is presented by Bakatschina et al., comparing kinematic variables between offensive and defensive wheelchair rugby wheelchairs in able-bodied participants. Perhaps surprisingly, they found that higher sprint velocities were achieved in defensive wheelchairs, indicating that that the higher performance observed in offensive vs. defensive wheelchair rugby players is a result of differences in disability, not wheelchair type. Staying within the sport of wheelchair rugby, Haydon et al. attempted to develop an algorithm to predict the impact of changing wheelchair setup on performance outcomes. Their on-court performance prediction was accurate for some, but less so for others, leading them to provide suggestions to improve accuracy further (e.g., inclusion of athlete activity limitations).

## Elite sports and performance, considerations related to Paralympic sports specific classification

Sound sports specific classification procedures are the basis for fair competition in Paralympic sports. Classification is an evolving field (as is the whole field of Paralympic sports), therefore adaptations to, or at times, completely new classification tests are required. Altman et al. investigated a test to determine arm coordination impairment (the spiral test). They found it useful and reliable to differentiate arm coordination impairment in people without impairment, making it a promising option for Paralympic classification.

The three other studies in the topic area of elite sport assess performance, and more specifically, how performance may be impacted by disability type and sport. Gee et al. provide an overview of the altered physiological response to exercise in disability and offer physiological considerations to benefit Paralympic performance, whilst highlighting research gaps. Gavel et al. address one of those gaps, namely the thermoregulatory response in National team wheelchair rugby players during international competition, relating thermal strain to movement time. Quittman et al. round this topic area off with a case report of a paratriathlete undergoing chronic myeloid leukaemia treatment, which dramatically reduced markers of physical capacity.

## Future perspectives

The articles in this special issue cover a range of approaches including experimental studies, systematic reviews, and a case study. Whilst broad in the topic areas covered, many provide evidence helping to maximise performance and/or minimise injury risk in sports suitable for manual wheelchair users. On the other hand, almost all presented articles show the continued need for research and are often only a starting point for gaining new knowledge about the multi-disciplinary impact of wheelchair sports on wheelchair users. Both the shown difficulty of measuring larger groups of participants, because of the relatively small and heterogeneous population, and the lack of strong longitudinal research designs hamper the level of evidence. One solution might be increased international collaboration between researchers, health and sports professionals, applying open science principles. Of course, at times this can be at odds with the competitive nature of top-level athletes. However, hesitancy to participate in such research may be overcome if overarching research questions are formulated with a goal to further professionalize adapted wheelchair sports as a whole. Buying into the research process by athletes, coaches and health professionals may further be facilitated by feeding back findings to the base, highlighting the relevance and applicability to the various stakeholders. In this context, the presentation of these 15 articles should be followed up with activities to engage lay audiences. Findings may be made palatable by presentations, summary videos or visual overviews aimed at specific target groups. Whatever the format, what unites the findings is their root in scientific principles. We are therefore grateful for this showcasing opportunity of the already high-quality research performed in this relative niche research area of adapted wheelchair sports—it certainly holds scope for further study.

